# Efficacy of the mHealth App Intellect in Improving Subclinical Obsessive-Compulsive Disorder in University Students: Randomized Controlled Trial With a 4-Week Follow-Up

**DOI:** 10.2196/63316

**Published:** 2024-12-16

**Authors:** Madeline Lee Yoon Li, Stephanie Lee Si Min, Oliver Sündermann

**Affiliations:** 1 National University of Singapore Singapore Singapore

**Keywords:** mobile health app, self-guided interventions, obsessive-compulsive disorder, cognitive behavioral therapy, maladaptive perfectionism, randomized controlled trial, behavioral, efficacy, mHealth app, university students, Singapore, symptoms

## Abstract

**Background:**

Obsessive-compulsive disorder (OCD) is the third most prevalent mental health disorder in Singapore, with a high degree of burden and large treatment gaps. Self-guided programs on mobile apps are accessible and affordable interventions, with the potential to address subclinical OCD before symptoms escalate.

**Objective:**

This randomized controlled trial aimed to examine the efficacy of a self-guided OCD program on the mobile health (mHealth) app Intellect in improving subclinical OCD and maladaptive perfectionism (MP) as a potential moderator of this predicted relationship.

**Methods:**

University students (N*=*225) were randomly assigned to an 8-day, self-guided app program on OCD (intervention group) or cooperation (active control). Self-reported measures were obtained at baseline, after the program, and at a 4-week follow-up. The primary outcome measure was OCD symptom severity (Obsessive Compulsive Inventory–Revised [OCI-R]). Baseline MP was assessed as a potential moderator. Depression, anxiety, and stress (Depression Anxiety and Stress Scales-21) were controlled for during statistical analyses.

**Results:**

The final sample included 192 participants. The intervention group reported significantly lower OCI-R scores compared with the active control group after the intervention (partial eta-squared [η*_p_*^2^]=0.031; *P*=.02) and at 4-week follow-up (η*_p_*^2^=0.021; *P*=.044). A significant, weak positive correlation was found between MP and OCI-R levels at baseline (*r*=0.28; *P*<.001). MP was not found to moderate the relationship between condition and OCI-R scores at postintervention (*P=*.70) and at 4-week follow-up (*P*=.88).

**Conclusions:**

This study provides evidence that the self-guided OCD program on the Intellect app is effective in reducing subclinical OCD among university students in Singapore. Future studies should include longer follow-up durations and study MP as a moderator in a broader spectrum of OCD symptom severity.

**Trial Registration:**

ClinicalTrials.gov NCT06202677; https://clinicaltrials.gov/study/NCT06202677

## Introduction

### Background

Obsessive-compulsive disorder (OCD) is one of the most common mental health disorders, with an estimated lifetime prevalence of 3.6% in Singapore [1] and from 2% to 3% globally [2]. OCD is characterized by the presence of recurrent unwanted thoughts, urges, or images (obsessions, compulsions, or both). Common contents of obsessions include preventing harm from mistakes or contamination; concern over order and incompleteness; and intrusive violent, religious, or sexual thoughts [3]. Typically, obsessions are ego-dystonic and inconsistent with one’s fundamental beliefs and personality. Hence, obsessions evoke significant anxiety or distress, which may drive the individual to carry out repetitive and excessive behaviors or mental acts (compulsions) that aim to neutralize the discomfort or avert the threat of a feared event.

OCD is associated with serious disability and decreased quality of life [4], leading to significant impairment in areas of family relations, social interactions, education, and work [5]. However, OCD-related impairment is not restricted to individuals who meet the full diagnostic criteria. OCD symptoms exist on a spectrum that varies in frequency and intensity across clinical and nonclinical populations [6]. Compared with the general population, individuals with subclinical OCD symptoms reported significantly lower life satisfaction and quality of life, and greater health care use [7]. In nonclinical populations, prevalence rates of intrusive thoughts have ranged from 74% to 99.4%, with individuals also found to carry out compulsive behaviors, such as checking, washing, or thought suppression [8,9]. Hence, recent research has emphasized early detection and intervention for individuals at risk of developing OCD [10].

The cognitive-behavioral model of OCD posits that obsessional distress stems from an individual’s appraisal of normal intrusive thoughts, images, and urges as extremely significant or threatening. Additionally, the individual appraises that the occurrence of the intrusion indicates their potential responsibility for causing harm [11]. These appraisals trigger distress and anxiety, which culminate in the decision to perform compulsions. Consequently, the short-term relief following compulsions negatively reinforces the behavior, leading to an increased behavioral tendency for compulsions over time [12]. As clinical obsessions develop from intrusive thoughts that most people experience [13], OCD symptoms in nonclinical samples are often investigated to uncover findings that can be extended to clinical populations.

The first-line psychotherapeutic treatment for OCD is cognitive behavioral therapy (CBT) with exposure and response prevention (ERP) [14]. ERP involves gradually confronting obsessions while refraining from compulsions, breaking the cycle of compulsions by teaching individuals to tolerate distress without engaging in counterproductive behaviors. ERP facilitates inhibitory learning, helping individuals form nonthreat associations that inhibit threat responses [15]. Extensive research has supported the superior efficacy of CBT in reducing OCD symptoms compared with placebo, relaxation therapy, and anxiety management [16,17]. The efficacy of CBT has been similarly demonstrated across countries, OCD severity, and treatment settings [18]. However, up to 50% of individuals drop out of treatment prematurely and approximately 25% refuse treatment [19]. Barriers to CBT include cost, limited availability of qualified practitioners, societal stigma, and difficulties attending sessions [20,21]. Hence, only a minority of individuals with OCD access evidence-based treatment [22].

Computerized internet-based CBT (ICBT) can reduce these barriers and overcome the limitations of face-to-face psychological interventions. Digital interventions are accessible from any location with an internet connection, offer a broad reach, are cost-effective, and are available 24/7 [23]. ICBT has demonstrated comparable effectiveness to face-to-face CBT for OCD [24], and self-guided ICBT has been suggested as an alternative to remote clinical support [25]. Self-guided ICBT has proven superior in reducing OCD symptom severity levels compared with waitlist and active control conditions [23,26]. Thus, self-guided digital interventions present a promising option for addressing the treatment gap and facilitating the delivery of evidence-based care.

While mobile health (mHealth) apps have gained traction in mental interventions for anxiety, stress, and depression [27], few studies are available on the efficacy of self-guided, mHealth app–based OCD programs that use CBT with ERP. Two randomized controlled trials (RCTs) examined the effectiveness of GG Relationships, Doubt, and Obsessions. However, as GG Relationships, Doubt, and Obsessions was designed to target symptoms of relationship OCD, the study samples were more selective, requiring all participants to have romantic relationship experience [28,29]. An RCT and an open pilot trial investigated the effectiveness of the apps OCfree and nOCD for OCD symptoms and found statistically significant reductions in OCD symptom severity at postintervention [30,31]. However, these apps were used alongside in-person therapy. Hence, it remains unclear whether self-guided mHealth apps for OCD are effective without therapist support. Moreover, previous studies focused on clinical OCD samples, potentially limiting generalizability to those with mild or subclinical symptoms who might benefit from low-intensity, self-guided treatments [32]. The authors also highlighted limitations, such as small sample sizes (N≤50), the lack of a control group not undergoing CBT, and the necessity for replication in future RCTs. Other limitations include the absence of follow-up assessments in 2 out of 4 studies and only 1 Asian sample being studied [29,31]. Notable gaps remain regarding the effectiveness of self-guided mHealth interventions for subclinical OCD populations.

Moreover, variables affecting the efficacy of self-guided mHealth app programs for OCD are underexplored. Only Gamoran and Doron [33] examined moderators in a sample of 46,955 users of OCD.app. Initial OCD severity, age, gender, trait mood, and app use data were identified as predictors of change. However, no psychological variables were analyzed as potential outcome moderators, leaving a gap in OCD treatment research [34]. A potential moderator of OCD outcomes from mHealth app programs is maladaptive perfectionism (MP). Perfectionism refers to one’s tendency to demand and pursue unrealistically high standards of performance, accompanied by excessively critical self-evaluations [35]. Frost et al [36] proposed a 6D measure of perfectionism, which involves being overly concerned about making mistakes (CM), setting and pursuing high personal standards, perceiving high parental expectations (PE) and parental criticism (PC), doubting the quality of one’s actions (DA), and preferring order and organization. As a multidimensional construct, perfectionism comprises adaptive and maladaptive aspects. While perfectionistic individuals seek excellence which may promote well-being [37], they may experience dysfunction when excessively fixating on mistakes and meeting external standards. MP is commonly observed in individuals with OCD [38]. It is a risk factor for OCD development and has been viewed as an essential predisposing trait [39]. Additionally, MP has been shown to impede OCD treatment [40]. As moderators can help to identify individuals who may face a poor prognosis and assist in matching individuals with appropriate treatments, it would be useful to examine how baseline MP levels can affect intervention outcomes for self-guided CBT-based mHealth app programs for OCD.

### This Study

Using an RCT design, we evaluated the efficacy of a self-guided program on an mHealth app in reducing subclinical OCD symptoms and severity among university students from Singapore. Additionally, we examined the association between MP and OCD symptom severity and investigated MP as a moderator of intervention outcomes. Although the mean age of onset for OCD is around 20 years, intervening in university students with subclinical symptoms can still prevent further progression. Early intervention, even at this stage, is crucial for reducing the risk of symptom escalation and long-term impairment.

Given that OCD ranks among the top 3 most prevalent mental health conditions in Singapore, with a lifetime prevalence that is approximately 0.4% to 1.6% higher than the global average, it is crucial to identify accessible and effective OCD interventions. In particular, Singaporeans aged 18 to 34 years have the highest lifetime prevalence rates of OCD, at 6.7% [1]. Yet, OCD remains poorly recognized and has the second-largest treatment gap out of at least 7 mental disorders studied in Singapore [41]. Given the typical onset of OCD in adolescence and early adulthood and its likely chronic course without appropriate intervention [42], it is critical for this population to access evidence-based interventions before symptoms escalate [1]. Young adults have responded positively to mHealth apps, citing their appeal due to flexibility, interactivity, and accessibility [43]. Furthermore, as a population with high smartphone use and common obstacles to accessing traditional mental health services, mHealth apps offer considerable promise to engage Singapore’s young adults [44,45].

Given Singapore’s cultural context, MP is also a relevant variable to examine. Extensive research has indicated that Asian populations generally exhibit significantly higher levels of perfectionism compared with African and White populations [46,47], which may be due to cross-cultural differences in parenting. Asian parents were found to set higher expectations for their children than European American parents [48] and provide conditional love based on their children’s achievements [49]. Additionally, Asian parents typically adopt more controlling and directive parenting styles compared with European parents [50], which is associated with the development of MP [51]. Thus, individuals who grew up in such demanding environments may develop perfectionism as they internalize these standards [52]. In a sample of 302 Singaporean children, a large proportion was found to exhibit high levels of MP [53]. This finding may be attributed to parental intrusiveness and the general emphasis on academic achievement in Singapore, which have been identified as factors that predict increased perfectionistic strivings and concerns [54,55]. Hence, as findings on OCD and MP may be culturally influenced, these variables are pertinent to study in Singapore for the development of effective interventions.

**Figure 1:**
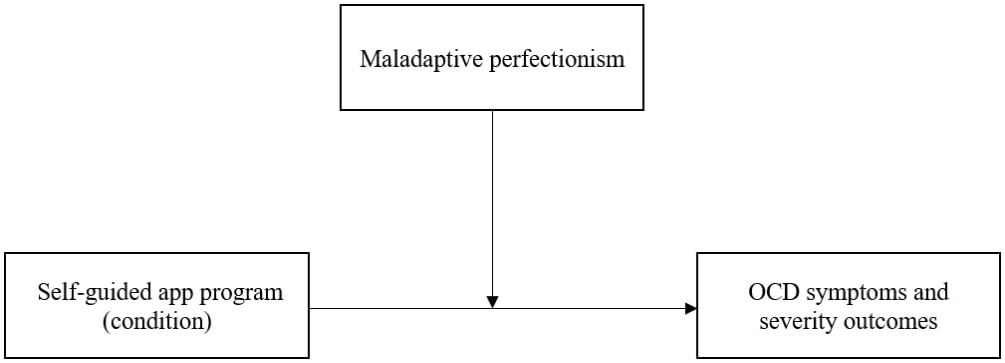
Predicted relationship with maladaptive perfectionism as a moderator of the direct effect of the self-guided app program on OCD symptom and severity outcomes. OCD: obsessive-compulsive disorder.

The hypotheses of the study are in 3 parts. First, we expected participants in the OCD program to report significant reductions in OCD outcomes compared with the active control group at postintervention and 4-week follow-up. Second, we hypothesized that baseline OCD symptom severity and MP will be positively correlated. Third, we predicted that participants with the lowest levels of MP would experience the greatest reduction in OCD symptoms and severity at postintervention and 4-week follow-up (Figure 1).

## Methods

### Design

This RCT used a 2×3 mixed factorial design with condition (intervention vs active control) as the between-subjects factor and time of assessment (baseline vs postintervention vs 4-week follow-up) as the within-subjects factor. The intervention group participated in an 8-day, self-guided OCD program, and the active control group participated in an 8-day, self-guided cooperation program. The dependent variable measured was OCD symptom severity. Additionally, general psychological distress was measured to be controlled for during analyses, and MP was measured for correlation and moderation analyses.

### Ethical Considerations

The study’s ethics was approved by the National University of Singapore’s (NUS) Institutional Review Board (NUS-IRB-2023-444). The study was also preregistered with ClinicalTrials.gov (registration NCT06202677). All study procedures adhered to the ethical standards outlined by the Declaration of Helsinki (as revised in 2000). Participants were provided with a detailed Participant Information Sheet, which explained the study's objectives, procedures, potential risks, and benefits. Written informed consent was obtained from all participants prior to their enrollment in the study, and they were explicitly informed of their right to withdraw at any point without penalty.

### Power Calculation

A meta-analysis examining low-intensity, technology-delivered CBT programs for OCD found small effect size reductions in OCD symptoms posttreatment [56]. Using Cohen *f*=0.10, α=0.05, and power=0.80, G*Power 3.1 revealed a minimum number of 164 participants. Attrition rates in similar mHealth app–based studies conducted among NUS students have ranged from 1% to 44.9% [57-59]. Additionally, accounting for 10% of invalid data typically encountered in web-based research studies [60], this study aimed to recruit a minimum of 206 participants.

### Recruitment and Study Participants

The sample of 225 participants had a mean age of 21.68 (SD 1.71) years and consisted of 171 female individuals, 52 male individuals, 1 nonbinary individual, and 1 individual of other gender. Recruitment was carried out by word of mouth and on the university recruitment platforms. Participants were reimbursed with either course credits or monetary cash (see the *Procedure* section below). The inclusion criteria required participants to be a Singapore citizen or permanent resident, be able to read and understand English, be a student from the NUS between the ages of 18 and 30 years, present with mild or subclinical OCD symptoms, and own a mobile phone to download the Intellect app for use in the study. Participants were also required to refrain from using external mental health services during their participation in the study. Individuals with moderate to high OCD symptom severity, as determined by their OCI-R (Obsessive Compulsive Inventory–Revised) scores, were excluded and directed to appropriate resources for the necessary treatment

### Materials

Intellect is an mHealth app that can be downloaded from the Apple App Store and the Google Play Store. It hosts numerous self-guided programs that have been reviewed by clinical psychologists and cognitive behavioral therapists. The app adheres to the highest standards of data security and privacy, using zero-knowledge encryption to ensure user data confidentiality. Various self-guided programs on the app have been validated in previous RCTs using samples of Singaporean university students: “Stress,” “Improving your Body Image,” and “Anxiety & Worry” [57-59]. In this study, participants used Intellect to access either the “Obsessive Compulsive Disorder” or “Cooperation” learning program, depending on their assigned condition.

### Learning Programs

#### OCD Program (Intervention Condition)

**Figure 2:**
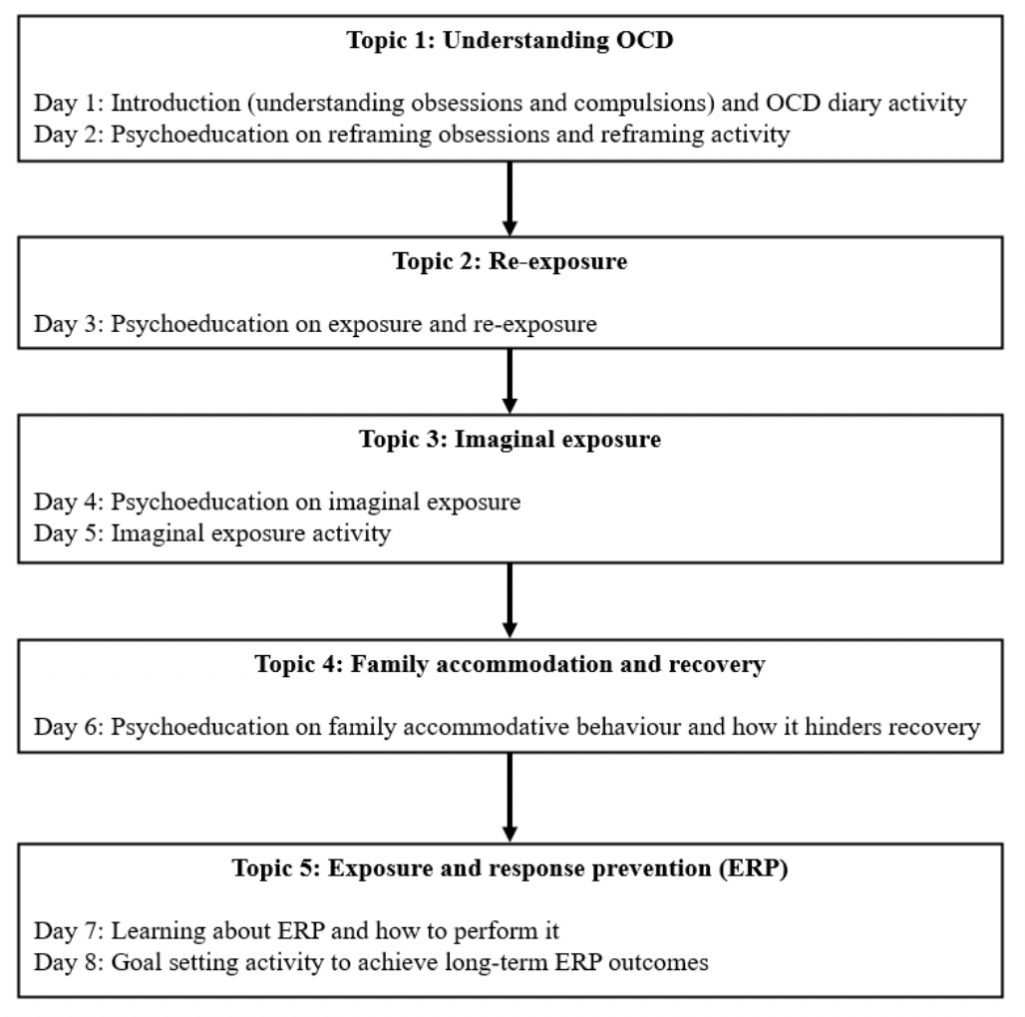
Timeline of OCD program. ERP: exposure and response prevention; OCD: obsessive-compulsive disorder.

This 8-day program used principles of CBT with ERP for OCD. Each daily session took approximately 5 minutes to complete. The program commenced with psychoeducation on obsessions and compulsions, and how to identify them. Subsequently, through a series of content learning and daily exercises that increase in difficulty, participants were guided to conduct exposure exercises while tolerating distress and refraining from compulsions. For example, in Topic 2 (Re-exposure), participants were asked to face their feared stimuli (eg, touching a “contaminated” surface) and refrain from performing compulsive behaviors (eg, excessive hand-washing) while observing and tolerating the discomfort that arose. This program structure aimed to enable participants to actively practice using the skills that were taught, while not overwhelming them. During the final program session, participants were guided to set goals for continual progress. Due to the challenging nature of ERP, the program also provided advice on concerns that are frequently encountered during ERP. An overview and timeline of the program are presented in Table 1 and Figure 2.

**Table 1:** Overview of obsessive-compulsive disorder (OCD) program.

Topics	Content
Topic 1: Understanding OCD	Identify personal obsessions and compulsions Identify when/where obsessions and compulsions arise Understand how the importance placed on obsessions causes distress Understand that obsessions are not based on reality Develop skills to reframe obsessions
Topic 2: Re-exposure	Introduce the concept of re-exposure Understand the function of exposure and re-exposure exercises Develop skills to carry out re-exposure Develop skills to tolerate obsessional distress
Topic 3: Imaginal exposure	Introduce the concept of imaginal exposure Develop skills to carry out imaginal exposure
Topic 4: Family accommodation and recovery	Introduce the concept of family accommodation Understand how family accommodative behaviors can hinder recovery Develop skills to identify family accommodative behaviors
Topic 5: Exposure and response prevention (ERP)	Introduce the concept of ERP Understand the importance of prioritizing long-term OCD reduction goals over short-term discomfort Develop skills to confront obsessions and resist compulsions Develop goal-setting skills for long-term ERP progress

#### Cooperation Program (Active Control Condition)

Participants in the active control group were allocated to an 8-day, self-guided program on cooperation, which was housed within the same Intellect app but offered a different module unrelated to OCD content. Having an active control group ensured that participants in both groups shared as many experiences as possible. This controlled for nonspecific factors and attentional effects, which could impact the study’s results [61]. The program aimed to improve participants’ collaborative skills and interpersonal wellness through a series of content learning and practice exercises, including activities reflective exercises on communication skills. To ensure that participants in both conditions spent a comparable amount of time and effort, the approximate duration for completing each daily session was matched to be 5 minutes.

### Procedure

Interested individuals signed up for the study by responding to the web-based advertisement via a link to the web-based survey hosted on Qualtrics. First, they completed a prescreening test comprising measures on OCD and demographics and were screened for eligibility according to the inclusion criteria. Eligible participants read the Participation Information Sheet, and after providing informed consent, completed measures on psychological distress, and perfectionism.

Thereafter, participants were randomly allocated to the intervention or active control condition, using computer-generated random numbers in a 1:1 ratio. To minimize potential confounding effects, participants were instructed not to use any other mHealth apps for the study duration. Additionally, to reduce demand characteristics, participants were not informed of the conditions’ functions or the study’s objective to evaluate the efficacy of the OCD program. Instead, participants were informed that the study examined well-being and the learning effectiveness of mental health–related topics using a smartphone app. Participants were then instructed to download the Intellect app, create an account, and access the program of their allocated condition. Participants in the intervention group underwent 8 days of the OCD program, while participants in the active control group underwent 8 days of the cooperation program. Participation in each program took approximately 5 minutes per day. To encourage study adherence, participants were sent a daily reminder to complete app activities via WhatsApp Messenger or Telegram Messenger. To verify program completion, participants sent a screenshot of their learning logs to the investigator.

**Figure 3:**
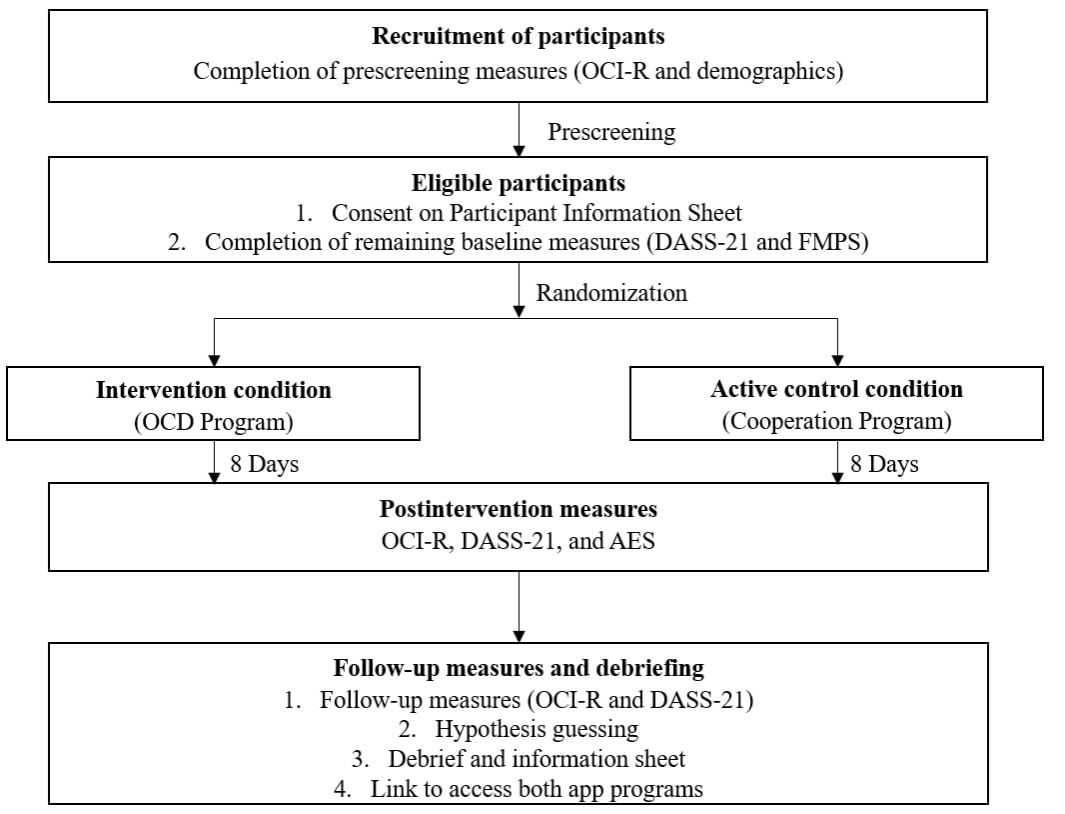
Study flowchart. AES: App Engagement Scale; DASS-21: Depression Anxiety Stress Scale-21; FMPS: Frost Multidimensional Perfectionism Scale; OCD: obsessive-compulsive disorder; OCI-R: Obsessive Compulsive Inventory–Revised.

After verification, participants were sent a postintervention survey link to complete the measures on OCD, psychological distress, and the App Engagement Scale (AES). Four weeks after program completion, participants were sent a follow-up survey link to complete measures on OCD and psychological distress. To further control for demand effects and confounders, participants were asked to guess the study’s hypothesis and indicate whether they had undergone mental health treatment during the past 4 weeks. Thereafter, they were debriefed on the study’s objectives and hypotheses. Participants who participated in the cooperation program were given access to the OCD program and vice versa. Participants were reimbursed with either SGD 10 (US $7.43) or 2 course credits after completion of the postintervention survey, and an additional SGD 2.50 (US $1.90) or 0.5 course credits after the follow-up survey. Figure 3 presents the study procedure.

### Measures

#### OCI-R Questionnaire

OCI-R [62] is an 18-item questionnaire that assesses OCD symptoms across 6 subscales: washing, checking, neutralizing, obsessing, ordering, and hoarding. Items are rated on a 5-point Likert scale (0=not at all; 4=extremely). OCD symptom severity can be derived by summing all the items to obtain a total score. Total OCI-R scores correspond to mild (OCI-R=0-15), moderate (OCI-R=16-27), and severe (OCI-R=28-72) OCD symptom severity [63]. In this study, the OCI-R was used as a measure of OCD symptom severity at baseline, postintervention, and 4-week follow-up. Participants with scores of 21 and above were screened out due to a likely presence of clinically significant OCD [62]. Subclinical OCD refers to above-average levels of symptoms that do not meet the clinical threshold but can still cause distress and functional impairment. Reducing even these subclinical symptoms is important to prevent potential escalation to more severe forms of OCD. The OCI-R showed acceptable internal consistency, with Cronbach α of 0.68.

#### Frost Multidimensional Perfectionism Scale

The Frost Multidimensional Perfectionism Scale (FMPS) [36] is a 35-item scale that assesses perfectionism across 6 subscales: CM, personal standards, PE, PC, DA, and preferring order and organization. Items are rated on a 5-point Likert scale (1=Strongly disagree; 5=Strongly agree). The FMPS has demonstrated good internal consistency and convergent validity with other measures of perfectionism within nonclinical and clinical samples [36]. In this study, the MP dimension (FMPS–Maladaptive Perfectionism [FMPS-MP]) was obtained by summing scores on the subscales of CM, DA, PE, and PC [64]. Perfectionism was assessed as a moderator of OCD symptom outcomes but was not directly addressed in the intervention content. The FMPS and FMPS-MP showed excellent internal consistency with Cronbach α of 0.91 and 0.92, respectively.

#### Depression Anxiety Stress Scale-21

The Depression Anxiety Stress Scale-21 (DASS-21) [65] is a set of 3 scales assessing depression, anxiety, and stress, and it is commonly used in student samples [66]. Each subscale contains 7 items, which are rated on a 4-point Likert scale (0=Did not apply to me at all; 3=Applied to me very much or most of the time). In this study, the DASS-21 total score was computed by summing the scores of all items and used as a measure of general psychological distress [65]. Additionally, as depression, stress, and anxiety have been identified in the pathogenesis and maintenance of OCD [67-69], baseline total DASS-21 scores were controlled for during analyses to disentangle the effect of the intervention on OCD symptoms from depression, stress, and anxiety. Internal consistency of the DASS-21 was excellent, with Cronbach α of 0.91.

#### AES Instrument

The AES [70] is a 7-item scale that assesses app engagement. Each item is rated on a 5-point Likert scale (1=definitely disagree; 5=definitely agree). The total score is derived by summing all the items. Internal consistency of the AES is good, with Cronbach α of 0.88.

### Analytic Approach

Incomplete responses and participants who indicated that they received psychotherapy during the study’s duration were excluded. Invalid data due to careless or insufficient effort responding were then sequentially identified in 2 steps and removed using a multiple hurdles approach [60]. First, response times of less than 500 seconds on the baseline survey, less than 215 seconds on the postintervention survey, and less than 180 seconds on the follow-up survey suggested that participants may have rushed through the questionnaires and app activities. Long string analysis and attention checks were carried out to confirm if these participants’ responses were invalid. Responses were identified and removed if the same option was selected across the entire scale of any measure or responses to attention-check items were incorrect. A total of 32 participants were excluded for failing these data quality checks, including response times indicating rushed responses, long string analysis, and attention check failures, as their data were deemed unreliable for analysis.

Finally, data were screened for univariate outliers that fell beyond 3 SDs from the mean. Winsorization was conducted to address these outliers by substituting them with the closest nonoutlying value [71]. Subsequently, all analyses were conducted with and without the outliers. As the results were consistent across both datasets, only results from the initial dataset were reported.

SPSS (version 26.0, IBM Corp) was used for all statistical analyses. Independent 2-tailed *t* tests, Mann-Whitney *U* tests, and chi-square tests were conducted to analyze differences between the intervention and active control groups on the baseline and demographic variables. While the initial intention-to-treat (ITT) analysis was conducted on the full dataset of 115 and 109 participants, 32 participants were excluded after failing data quality checks. Thus, the final numbers analyzed were 94 and 98, reflecting a modified per-protocol analysis after quality checks.

Missing data were assessed for attrition biases using independent 2-tailed *t* tests, chi-square tests, and Mann-Whitney *U* tests to ensure there were no significant differences between participants who completed all assessments and those who did not. As all missing data were verified to be missing at random, they were addressed using ITT analyses [72], whereby missing data on the postintervention and follow-up assessment were substituted with data from the last completed questionnaire. Missing AES data were replaced using the mean score.

### Preliminary Analyses

#### Main Analyses

Analysis of covariance (ANCOVA) examined if changes in OCD symptom severity at postintervention and follow-up were significantly different between the intervention and active control groups. ANCOVA is recommended for inferential testing intervention effects [73], as controlling for baseline scores ensures that results at postintervention and follow-up are attributable to the intervention [74]. ANCOVA was conducted on postintervention and follow-up OCI-R scores, with baseline OCI-R and DASS-21 scores as the covariates. The α level was set at *P*<.05. Cohen [75] guidelines for eta square were used to interpret effect sizes (η*_p_*^2^), whereby 0.01 to 0.05 indicates a small effect, 0.06 to 0.13 indicates a moderate effect, and 0.14 and above indicates a large effect.

#### Correlation and Moderation Analyses

Pearson correlation was conducted to test if baseline OCI-R scores were correlated with FMPS-MP scores. Due to nonnormality of baseline OCI-R scores, the bias-corrected and accelerated method (BCa) with 1000 bootstrap iterations was used to generate 95% CIs for Pearson correlation coefficients [76]. Pearson correlation analysis with BCa is robust to nonnormality and retains type I error control by generating more accurate CIs [77,78].

Subsequently, Hayes PROCESS (version 4.2, IBM Corp, macro model 1) was used for moderation analyses. A total of 2 models were analyzed. Postintervention and follow-up OCI-R scores were used as the dependent variables in the first and second models, respectively. Each model ran a simple moderation analysis to investigate the moderating effects of MP on the relationship between condition and OCI-R scores at postintervention or follow-up. Baseline OCI-R and DASS-21 scores were entered as the covariate for all analyses. The 95% CIs were used, with 5000 bootstrap iterations. The interaction term between condition and FMPS-MP was interpreted, whereby *P*<.05 indicates that MP moderates OCI-R scores at postintervention or follow-up.

## Results

### Participant Characteristics

**Figure 4:**
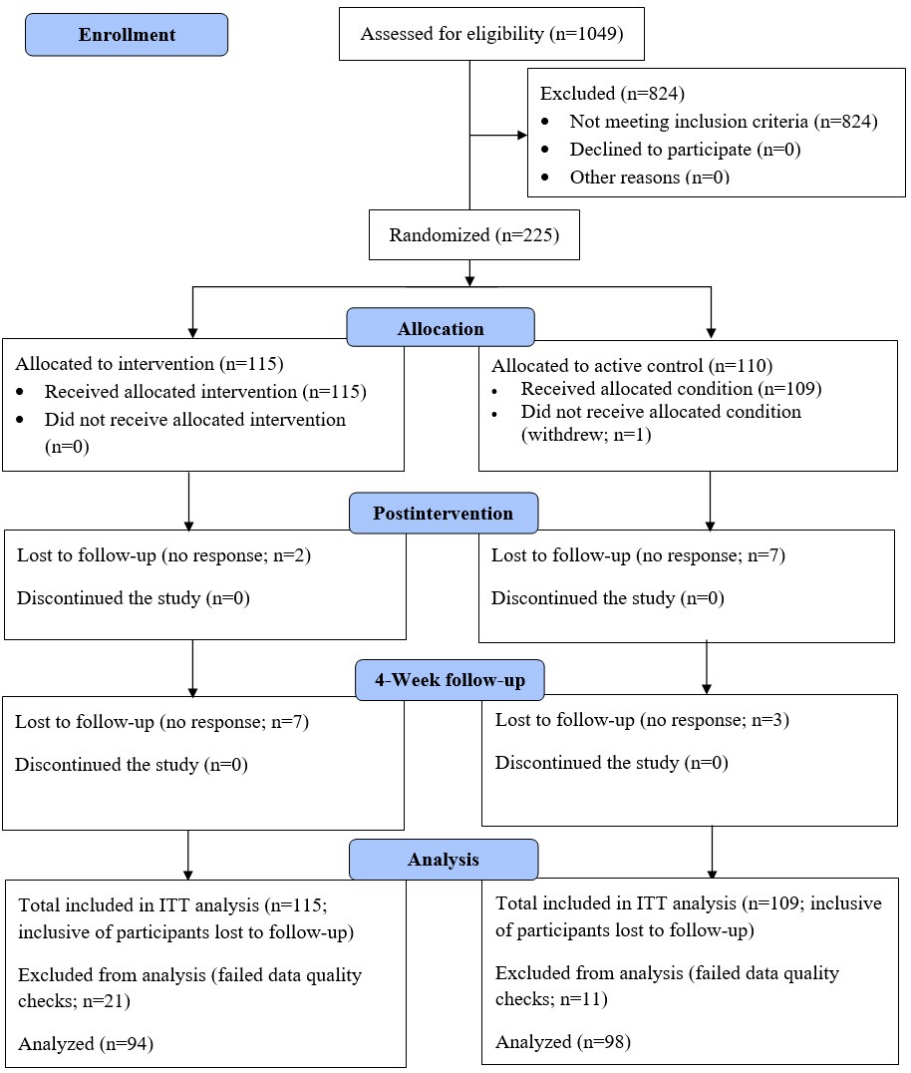
CONSORT (Consolidated Standards of Reporting Trials) flow diagram. ITT: Intention to treat.

A total of 1049 responses were screened for eligibility. Of these, 225 participants met the inclusion criteria, completed the baseline survey, and were randomly assigned to either the intervention or active control condition. One participant withdrew from the study before completing the postintervention assessment, resulting in 224 participants who completed the intervention. Among these, 215 (95.56%) participants completed the postintervention assessment, and 204 (90.67%) participants completed the follow-up assessment. Additionally, 32 participants were excluded from the analyses after failing data quality checks, leading to a final sample of 192 participants included in the statistical analyses (Figure 4).

At baseline, 111 participants exhibited mild OCD symptom levels, and 81 participants exhibited moderate OCD symptom levels. Psychological distress was measured using the DASS-21, with average scores at baseline (mean 11.45, SD 8.66), postintervention (mean 9.93, SD 8.37), and follow-up (mean 11.59, SD 9.46), indicating that the participants were generally experiencing mild distress throughout the study.

### Preliminary Analyses

The intervention and active control groups did not differ significantly on baseline demographic and outcome variables (Tables 2 and 3).

**Table 2:** Descriptive statistics for demographic variable of gender by condition.

Demographic variable			Full sample (n=192), n (%)		Intervention condition (n=94), n (%)		Active control condition (n=98), n (%)		*P* value	
**Gender**										.47
	Women	147 (76.6)		74 (78.7)		73 (74.5)				
	Men	43 (22.4)		19 (20.2)		24 (24.5)				
	Nonbinary	1 (0.5)		1 (1.1)		0 (0.0)				
	Other	1 (0.5)		0 (0.0)		1 (1.0)				

**Table 3:** Descriptive statistics for baseline demographic and outcome variables by condition (n=192).

Variable	Intervention condition (n=94), mean (SD)	Active control condition (n=98), mean (SD)	*P* value
FMPS-MP^a^	61.21 (14.56)	57.93 (12.60)	.10
Age (years)	21.69 (1.46)	21.74 (1.82)	.82
OCI-R^b^	12.83 (5.41)	13.01 (5.18)	.86
DASS-21^c^	11.83 (9.32)	11.09 (8.00)	.79

^a^FMPS-MP: Frost Multidimensional Perfectionism Scale–Maladaptive Perfectionism.

^b^OCI-R: Obsessive Compulsive Inventory–Revised.

^c^DASS-21: Depression Anxiety Stress Scale-21.

For participants lost to postintervention and follow-up, Mann-Whitney *U* tests, chi-square tests, and independent 2-tailed *t* tests revealed no attrition-related biases across demographic and outcome variables. ITT analysis was conducted to address participants’ missing data by bringing forward scores from their last completed survey. Mean substitution was conducted to address missing AES scores.

Independent 2-tailed *t* tests indicated that participants in both conditions enjoyed using the app, with no significant differences (*P*=.81) found between the intervention group (mean 25.33, SD 3.90) and the active control group (mean 25.47, SD 4.01) on the AES.

Participants were directly asked after the study if they believed they could guess the hypothesis. Mann-Whitney *U* tests indicated that participants who correctly guessed the study’s hypothesis did not differ significantly from participants who incorrectly guessed the hypothesis on all measures at postintervention and follow-up.

### Main Analyses

The assumptions for ANCOVA were met, except for the assumption for normality. However, due to the robustness of ANCOVA against deviations from normality when sample sizes are larger than 100 [77,78], analysis using ANCOVA proceeded. At both postintervention and follow-up, the intervention group reported significantly lower OCI-R scores compared with the active control group, with a small effect size (Table 4).

**Table 4:** Means, SDs, univariate *F* values, and effect sizes for OCI-R^a^ scores at postintervention and 4-week follow-up.

	Intervention, mean (SD)	Control, mean (SD)	*F* test (*df*)	*P* value	ES^b^
Baseline	12.83 (5.41)	13.01 (5.18)	—^c^	—	—
Postintervention	11.03 (7.40)	13.04 (6.53)	5.79^d^ (1)	.02^e^	.030
Follow-up	10.53 (7.36)	12.37 (7.46)	4.11^d^ (1)	—	—

^a^OCI-R: Obsessive Compulsive Inventory–Revised.

^b^Effect size of 0.01=small, 0.06=moderate, 0.14=large [75].

^c^Not applicable.

^d^*P*<.05.

^e^Significant *P* value at .05.

### Correlation and Moderation Analyses

Results from Pearson correlation analysis revealed that OCI-R and FMPS-MP were significantly correlated (*P*<.001) with a weak positive correlation (*r*=.28), and the BCa bootstrap (95% CI 0.14-0.41) did not include zero.

Moderation analyses indicated a significant effect in the overall moderation model with OCI-R scores as the dependent variable and FMPS-MP as the moderator. At postintervention, the model showed *R*^2^=0.38, *F*_5,186_=22.57, and *P*<.001, and at follow-up, it showed *R*^2^=0.29, *F*_5,186_=15.23, and *P*<.001. However, the interaction term between the condition and FMPS-MP was not statistically significant at postintervention and follow-up. Results are displayed in Table 5.

**Table 5:** Standardized coefficients, SEs, t values, *P* values, and 95% CIs for moderation analyses.

Model and variable			OCI-R^a^				
		β		SE	*P* value	*t*	95% CI
**1 (T1^b^-T2^c^)**							
	Constant	4.85		5.93	.41	0.82	–6.85 to 16.56
	Condition	–3.49		3.69	.34	–0.95	–10.77 to 3.78
	FMPS-MP^d^	0.01		0.10	.95	0.07	–0.19 to 0.21
	Condition×FMPS-MP	0.02		0.06	.70	0.39	–0.10 to 0.14
	DASS-21^e^	0.08		0.05	.12	1.55	–0.02 to 0.18
	OCI-R	0.70		0.08	<.001^f^	8.19	0.53 to 0.86
**2 (T1-T3^g^)**							
	Constant	–0.51		6.72	.94	–0.077	–13.77 to 12.74
	Condition	–1.65		4.18	.69	–0.39	–9.88 to 6.59
	FMPS-MP	0.14		0.11	.23	1.21	–0.09 to 0.36
	Condition×FMPS-MP	–0.01		0.07	.88	–0.16	–0.15 to 0.12
	DASS-21	0.16		0.06	.01^h^	2.79	0.05 to 0.28
	OCI-R	0.29		0.10	<.001^f^	4.26	0.22 to 0.60

^a^OCI-R: Obsessive Compulsive Inventory–Revised.

^b^T1: preintervention.

^c^T2: postintervention.

^d^FMPS-MP: Frost Multidimensional Perfectionism Scale–Maladaptive Perfectionism.

^e^DASS-21: Depression Anxiety Stress Scale-21.

^f^*P* <.001.

^g^T3: follow-up.

^h^*P*<.05.

## Discussion

### Principal Findings

This study evaluated the efficacy of an 8-day, self-guided OCD program on an mHealth app compared with active control in reducing subclinical OCD symptoms and severity in a sample of Asian university students. The study further investigated the correlation between MP and OCD symptomatology, and MP as a factor that might help identify individuals who will benefit most from the self-guided program. The study addresses important gaps in the literature regarding the dearth of RCTs studying evidence-based mHealth app programs for OCD and the factors that influence the efficacy of such programs.

Our hypotheses regarding the efficacy of the app-based OCD program were supported. The intervention group reported significant reductions in OCD symptom severity compared with the active control group at both postintervention and follow-up. Consistent with past studies on longer ICBT or in-person interventions, our findings revealed that an 8-day, self-guided program using principles of CBT with ERP can reduce OCD symptoms and severity [16,23,79]. The small effect size reductions for OCD symptom severity in our OCD program are also comparable with a 12-week self-guided ICBT program for OCD at postintervention [80]. Taken together, these findings suggest that OCD gains can be obtained in a shorter duration through self-guided mHealth app programs. However, as there is limited research regarding the optimal duration of self-guided interventions, future research should examine how the efficacy of self-guided, low-intensity interventions for OCD might vary with program duration. While longer interventions may offer more comprehensive content and exercises, shorter interventions may offer increased convenience and improve program completion rates.

Our study also extended the findings from guided, 6-week-long, app-based program by Hwang et al [31], demonstrating that even without therapist support, app-based CBT can be beneficial for individuals with OCD symptoms. However, compared with the study by Hwang et al [31], which used a sample with clinical OCD, our study’s subclinical sample did not yield large effect size reductions for OCD. Despite our study’s overall small effect sizes, this result is larger or comparable to effect sizes found after using the self-guided OCD.app for approximately 8.52 days. Specifically, Gamoran and Doron [33] found null effect sizes for participants with mild OCD symptom levels, and small effect sizes for participants with moderate OCD symptom levels. Research from previous RCTs has suggested that mHealth app use leads to greater decreases in symptoms for participants with moderate to severe OCD symptom levels [81], compared with those with less severe OCD symptom levels [29]. As such, because our study’s sample comprised participants with both mild and moderate OCD symptom severity, it is possible that analyzing their data collectively diluted the effect size. Moreover, participants with OCI-R scores near zero were included in our study. Such participants would expectedly have a limited reduction in OCD symptom severity after the intervention. As participants in both groups generally reported low OCI-R scores at baseline, it is possible that a floor effect limited intervention effects. Hence, it would be fruitful for future RCTs to investigate how effect sizes may vary across different levels of OCD symptom severity after using self-guided app-based OCD programs. Additionally, the efficacy of such programs should be tested in samples with clinically significant levels of OCD symptom severity to better understand their clinical utility.

Overall, these findings provide preliminary support for the use of CBT with ERP on mHealth apps to reduce OCD symptom severity in Asian university students. As such, our findings align with the National Institute for Health and Care Excellence [32] stepped-care approach for managing OCD-related symptoms using low-intensity treatments. Additionally, the 4-week follow-up effects were encouraging as they suggest that the app-based OCD program can produce sustained effects, addressing the lack of follow-up data in mHealth app research [82]. As ICBT for OCD has been found to produce treatment effects maintained after 2 years [83], future mHealth app studies for OCD may seek to administer longer follow-up assessments to evaluate the long-term maintenance of treatment gains.

Our hypothesis that MP predicts OCD symptom severity was also supported. This finding corroborated with prior research that MP is highly prevalent in individuals with OCD [38]. Based on the cognitive-behavioral model of OCD, exhibiting higher levels of MP increases the risk of OCD as individuals are prone to beliefs that there is a perfect method to performing any task (eg, “I must order my books perfectly parallel to each other”). Additionally, individuals with high MP often have an inflated sense of responsibility over obsessions, anticipating that small mistakes and failures will have catastrophic consequences which they are responsible for preventing [84]. Consequently, obsessive distress and anxiety drive the individual to perform compulsions to dispel the threat of their obsessions [85]. However, the strength of the correlation found between MP and OCD symptoms in our study was weaker than that of prior research [86]. This may be due to a range restriction of OCI-R scores. Given that participants with clinically significant levels of OCD were excluded from our sample, our study’s participants represent a narrower segment of the OCD severity spectrum than the general, broader OCD population. By limiting the variability in OCD symptom severity, the true strength of the correlation between MP and OCD symptom severity may have been obscured [87].

Furthermore, our hypothesis that MP would moderate the decrease of OCD symptoms at postintervention and follow-up was not supported. This result was unexpected, given that MP has been found to impede OCD treatment [40,88]. The weak correlation between MP and OCD symptom severity may have consequently led to and explained the lack of moderation effect of MP on the decrease of OCD symptoms at postintervention and follow-up. Another possible explanation for the lack of significant moderation effect could be due to the sample’s high mean MP and small spread (mean 59.46, SD 13.72) [86,89]. As such, there might have been insufficient variability in MP scores needed to detect a moderating effect. Future studies may consider further examining MP as a moderator in samples with a wider range of OCD symptom severity.

### Strengths and Limitations

The RCT design enabled the establishment of causal conclusions from the study’s intervention and results [90]. Additionally, using an active control group controlled for attentional influences and nonspecific variables. Our study also had an attrition rate of 9.33%, which is much lower than participant dropout rates of up to 44.44% in similar studies on app-based OCD programs [81]. Furthermore, no attrition-related biases were detected in participants who discontinued the study. Altogether, these factors strengthened the overall validity of our results.

However, our study had several limitations. First, the low attrition and relatively high compliance observed may be partially attributable to the daily messaging by the study team, which is unlikely to be replicable in real-world settings. Next, data regarding how long participants spent applying skills from the programs in their daily lives were not collected. CBT research has found that greater application of CBT skills improves treatment outcomes [91], hence it is probable that participants who practiced the skills taught in our programs more frequently would have made increased gains. Moreover, there was no objective assessment of participant engagement and effort expended on the intervention programs as the time participants spent using the app was not tracked or controlled for. However, as poor engagement with mHealth apps has been associated with reduced gains [92], measures were taken to encourage adherence and ensure that app activities were fully completed. Participants received daily reminders to complete the app activities, and the app was programmed such that participants could only proceed to the next session if all preceding sessions were completed. Crucially, all participants were verified to have completed their assigned program. Given that no significant differences were found on the AES, both groups likely shared similar levels of engagement and time spent on the programs. Regardless, future studies should control for the duration of mHealth app use and investigate how the duration of app engagement may impact treatment outcomes.

Next, self-report measures are susceptible to individual biases, which could have contributed to the study’s results. Social desirability bias is a concern because participant blinding was not fully feasible, given that the study’s hypothesis might be guessed based on the content of the allocated program. However, participants who correctly guessed the study’s hypothesis did not report significantly different results on OCD outcomes compared with incorrect participants, which gives us some confidence that the effect of social desirability bias was limited. Nonetheless, our results should be interpreted with caution. Retrospective recall biases may also be present as the OCI-R required participants to report their experiences in the past month. To strengthen the accuracy of participants’ self-reports on treatment outcomes, future studies may use daily journaling methods which can minimize memory distortion from prolonged recall periods [93].

Finally, as participants were recruited from one university and comprised mostly of female students, the external validity of our study may be limited. Future studies may replicate our findings with a more diverse sample of young adults.

In sum, the Intellect app offers a valuable solution for early intervention in subclinical OCD, helping prevent the escalation of symptoms into more severe forms. Its self-guided, CBT-based structure provides accessible support without the need for therapist involvement, addressing gaps in traditional mental health services. By offering a scalable and cost-effective option, the app enables individuals to manage their mental health more effectively, improving quality of life and reducing the risk of future clinical impairment.

### Conclusion

Overall, our study provides preliminary support for the efficacy of a self-guided mHealth app program using principles of CBT with ERP in improving OCD symptom severity in young adults, producing gains maintained after 4 weeks. While MP was found to predict OCD symptom severity, MP did not moderate treatment outcomes. Identifying moderators can optimize outcome and treatment delivery on OCD-related mHealth apps. Hence, the impact of MP on treatment outcomes should be further studied in a broader spectrum of OCD symptom severity. Given the potential for cost-effective and accessible mHealth app programs to reach young adults and overcome traditional treatment barriers, future studies should aim to improve the efficacy of these programs.

## Appendix

Multimedia Appendix 1 CONSORT-eHEALTH checklist (V 1.6.2).

## Abbreviations

AES: App Engagement Scale
ANCOVA: analysis of covariance
BCa: bias-corrected and accelerated method
CBT: cognitive behavioral therapy
CM: concerned about making mistakes
DA: doubting the quality of one’s actions
DASS-21: Depression Anxiety Stress Scale-21
ERP: exposure and response prevention
FMPS-MP: Frost Multidimensional Perfectionism Scale
FMPS-MP: Frost Multidimensional Perfectionism Scale–Maladaptive Perfectionism
ICBT: internet-based CBT
ITT: intention-to-treat
mHealth: mobile health
MP: maladaptive perfectionism
NUS: National University of Singapore
OCD: obsessive-compulsive disorder
OCI-R: Obsessive Compulsive Inventory–Revised
PC: parental criticism
PE: parental expectations
RCT: randomized controlled trial
