# Supplementary material for: Efficacy of the mHealth App Intellect in Improving Subclinical Obsessive-Compulsive Disorder in University Students: Randomized Controlled Trial With a 4-Week Follow-Up
Source: JMIR Mhealth Uhealth. 2024 Dec 16;12:e63316. doi: 10.2196/63316 (PMC11686029; doi:10.2196/63316)
Supplement: Multimedia Appendix 1 [file mhealth_v12i1e63316_app1.pdf]

b) to form a basis for appraisal of an ehealth trial (in terms of validity)

CONSORT-EHEALTH items/subitems are MANDATORY reporting items for studies published in the Journal of Medical Internet Research and other journals / scientific societies endorsing the checklist.

Items numbered 1., 2., 3., 4a., 4b etc are original CONSORT or CONSORT-NPT (non-pharmacologic treatment) items.
Items with Roman numerals (i., ii, iii, iv etc.) are CONSORT-EHEALTH extensions/clarifications.

As the CONSORT-EHEALTH checklist is still considered in a formative stage, we would ask that you also RATE ON A SCALE OF 1-5 how important/useful you feel each item is FOR THE PURPOSE OF THE CHECKLIST and reporting guideline (optional).

Mandatory reporting items are marked with a red *.
In the textboxes, either copy & paste the relevant sections from your manuscript into this form - please include any quotes from your manuscript in QUOTATION MARKS,
or answer directly by providing additional information not in the manuscript, or elaborating on why the item was not relevant for this study.

YOUR ANSWERS WILL BE PUBLISHED AS A SUPPLEMENTARY FILE TO YOUR PUBLICATION IN JMIR AND ARE CONSIDERED PART OF YOUR PUBLICATION (IF ACCEPTED).
Please fill in these questions diligently. Information will not be copyedited, so please use proper spelling and grammar, use correct capitalization, and avoid abbreviations.

DO NOT FORGET TO SAVE AS PDF _AND_ CLICK THE SUBMIT BUTTON SO YOUR ANSWERS ARE IN OUR DATABASE !!!

Citation Suggestion (if you append the pdf as Appendix we suggest to cite this paper in the caption):
Eysenbach G, CONSORT-EHEALTH Group
CONSORT-EHEALTH: Improving and Standardizing Evaluation Reports of Web-based and Mobile Health Interventions
J Med Internet Res 2011;13(4):e126

Madeline Lee

Primary Affiliation (short), City, Country *

University of Toronto, Toronto, Canada

National University of Singapore

Your e-mail address *

abc@gmail.com

madelinelee@u.nus.edu

Title of your manuscript *

Provide the (draft) title of your manuscript.

Efficacy of the Mobile Health App Intellect in Improving Subclinical Obsessive Compulsive Disorder in University Students: Randomized Controlled Trial with a 4-Week Follow-up

Language(s) *

What language is the intervention/app in? If multiple languages are available, separate by comma (e.g. "English, French")

English

URL of your Intervention Website or App

e.g. a direct link to the mobile app on app in appstore (itunes, Google Play), or URL of the website. If the intervention is a DVD or hardware, you can also link to an Amazon page.

https://apps.apple.com/us/app/intellect-create-a-better-you/id1483308512

URL of an image/screenshot (optional)

Your answer

Primary Medical Indication/Disease/Condition *

e.g. "Stress", "Diabetes", or define the target group in brackets after the condition, e.g. "Autism (Parents of children with)", "Alzheimers (Informal Caregivers of)"

Obsessive compulsive disorder

Primary Outcomes measured in trial *

comma-separated list of primary outcomes reported in the trial

obsessive compulsive behavior (OCI-R)

Secondary/other outcomes

Are there any other outcomes the intervention is expected to affect?

Depression, anxiety, stress (DASS-21), app engagement (AES), maladaptive perfectionism (FMPS)

Approx. Percentage of Users (starters) still using the app as recommended after 3 months *

- ○ unknown / not evaluated
- ○ 0-10%
- ○ 11-20%
- ○ 21-30%
- ○ 31-40%
- ○ 41-50%
- ○ 51-60%
- ○ 61-70%
- ○ 71%-80%
- ○ 81-90%
- ○ 91-100%
- ◉ Other: App program ends after 8 days

Article Preparation Status/Stage *

At which stage in your article preparation are you currently (at the time you fill in this form)

- ( ) not submitted yet - in early draft status
- ( ) not submitted yet - in late draft status, just before submission
- (•) submitted to a journal but not reviewed yet
- ( ) submitted to a journal and after receiving initial reviewer comments
- ( ) submitted to a journal and accepted, but not published yet
- ( ) published
- ( ) Other:

- ○ JMIR Public Health
- ○ JMIR Formative Research
- ○ Other JMIR sister journal
- ○ Other:

Is this a full powered effectiveness trial or a pilot/feasibility trial? *

- ○ Pilot/feasibility
- ◉ Fully powered

Manuscript tracking number *

If this is a JMIR submission, please provide the manuscript tracking number under "other" (The ms tracking number can be found in the submission acknowledgement email, or when you login as author in JMIR. If the paper is already published in JMIR, then the ms tracking number is the four-digit number at the end of the DOI, to be found at the bottom of each published article in JMIR)

- ○ no ms number (yet) / not (yet) submitted to / published in JMIR
- ◉ Other: ms#63316

Other:

1a-i) Identify the mode of delivery in the title

Identify the mode of delivery. Preferably use “web-based” and/or “mobile” and/or “electronic game” in the title. Avoid ambiguous terms like “online”, “virtual”, “interactive”. Use “Internet-based” only if Intervention includes non-web-based Internet components (e.g. email), use “computer-based” or “electronic” only if offline products are used. Use “virtual” only in the context of “virtual reality” (3-D worlds). Use “online” only in the context of “online support groups”. Complement or substitute product names with broader terms for the class of products (such as “mobile” or “smart phone” instead of “iphone”), especially if the application runs on different platforms.

	1	2	3	4	5	
subitem not at all important	○	○	○	○	◉	essential

Clear selection

Does your paper address subitem 1a-i? *

Copy and paste relevant sections from manuscript title (include quotes in quotation marks "like this" to indicate direct quotes from your manuscript), or elaborate on this item by providing additional information not in the ms, or briefly explain why the item is not applicable/relevant for your study

"Mobile Health App Intellect"

[illegible]

Copy and paste relevant sections from manuscript title (include quotes in quotation marks "like this" to indicate direct quotes from your manuscript), or elaborate on this item by providing additional information not in the ms, or briefly explain why the item is not applicable/relevant for your study

Item is not applicable as our study is evaluating a mobile app program, all components are mobile app based

1a-iii) Primary condition or target group in the title

Mention primary condition or target group in the title, if any (e.g., “for children with Type I Diabetes”) Example: A Web-based and Mobile Intervention with Telephone Support for Children with Type I Diabetes: Randomized Controlled Trial

	1	2	3	4	5	
subitem not at all important	○	○	○	○	◉	essential

Clear selection

and blinding status.

1b-i) Key features/functionalities/components of the intervention and comparator in the METHODS section of the ABSTRACT

Mention key features/functionalities/components of the intervention and comparator in the abstract. If possible, also mention theories and principles used for designing the site. Keep in mind the needs of systematic reviewers and indexers by including important synonyms. (Note: Only report in the abstract what the main paper is reporting. If this information is missing from the main body of text, consider adding it)

	1	2	3	4	5	
subitem not at all important	○	○	○	○	◉	essential

Clear selection

1b-ii) Level of human involvement in the METHODS section of the ABSTRACT

Clarify the level of human involvement in the abstract, e.g., use phrases like “fully automated” vs. “therapist/nurse/care provider/physician-assisted” (mention number and expertise of providers involved, if any). (Note: Only report in the abstract what the main paper is reporting. If this information is missing from the main body of text, consider adding it)

	1	2	3	4	5	
subitem not at all important	○	○	○	○	◉	essential

Clear selection

Does your paper address subitem 1b-ii?

Copy and paste relevant sections from the manuscript abstract (include quotes in quotation marks "like this" to indicate direct quotes from your manuscript), or elaborate on this item by providing additional information not in the ms, or briefly explain why the item is not applicable/relevant for your study

"Methods: University students (N=225) were randomized to an 8-day self-guided app program on OCD (intervention group) or cooperation (active control). Self-reported measures were obtained at baseline, after the program, and at 4-week follow-up. "

	1	2	3	4	5	
subitem not at all important	○	○	○	○	◉	essential

Clear selection

Does your paper address subitem 1b-iii?

Copy and paste relevant sections from the manuscript abstract (include quotes in quotation marks "like this" to indicate direct quotes from your manuscript), or elaborate on this item by providing additional information not in the ms, or briefly explain why the item is not applicable/relevant for your study

"Methods: University students (N=225) were randomized to an 8-day self-guided app program on OCD (intervention group) or cooperation (active control). Self-reported measures were obtained at baseline, after the program, and at 4-week follow-up."

Does your paper address subitem 1b-iv?

Copy and paste relevant sections from the manuscript abstract (include quotes in quotation marks "like this" to indicate direct quotes from your manuscript), or elaborate on this item by providing additional information not in the ms, or briefly explain why the item is not applicable/relevant for your study

"Results: The final sample included 192 participants."

1b-v) CONCLUSIONS/DISCUSSION in abstract for negative trials

Conclusions/Discussions in abstract for negative trials: Discuss the primary outcome - if the trial is negative (primary outcome not changed), and the intervention was not used, discuss whether negative results are attributable to lack of uptake and discuss reasons. (Note: Only report in the abstract what the main paper is reporting. If this information is missing from the main body of text, consider adding it)

	1	2	3	4	5	
subitem not at all important	○	○	○	○	●	essential

Clear selection

INTRODUCTION

2a) In INTRODUCTION: Scientific background and explanation of rationale

2a-i) Problem and the type of system/solution

Describe the problem and the type of system/solution that is object of the study: intended as stand-alone intervention vs. incorporated in broader health care program? Intended for a particular patient population? Goals of the intervention, e.g., being more cost-effective to other interventions, replace or complement other solutions? (Note: Details about the intervention are provided in “Methods” under 5)

	1	2	3	4	5	
subitem not at all important	○	○	○	○	◉	essential

Clear selection

before symptoms escalate [1]. Young adults have responded positively to mHealth apps, citing their appeal due to flexibility, interactivity, and accessibility [44]. Furthermore, as a population with high smartphone usage and common obstacles to accessing traditional mental health services, mHealth apps offer considerable promise to engage Singapore's young adults [45,46].

Given Singapore's cultural context, MP is also a relevant variable to examine. Extensive research has indicated that Asians generally exhibit significantly higher levels of perfectionism compared to Africans and Caucasians [47,48], which may be due to cross-cultural differences in parenting. Asian parents were found to set higher expectations for their children than European American parents [49] and provide conditional love based on their children's achievements [50]. Additionally, Asian parents typically adopt more controlling and directive parenting styles compared to European parents [51], which is associated with the development of MP [52]. Thus, individuals who grew up in such demanding environments may develop perfectionism as they internalize these standards [53]. In a sample of 302 Singaporean children, a large proportion was found to exhibit high levels of MP [54]. This finding may be attributed to parental intrusiveness and the general emphasis on academic achievement in Singapore which have been identified as factors that predict increased perfectionistic strivings and concerns [55,56]. Hence, as findings on OCD and MP may be culturally influenced, these variables are pertinent to study in Singapore for the development of effective interventions.

The hypotheses of the study are in three parts. First, we expected participants in the OCD program to report significant reductions in OCD outcomes compared to the active control group at post-intervention and 4-week follow-up. Second, we hypothesized that baseline OCD symptom severity and MP will be positively correlated. Third, we predicted that participants with the lowest levels of MP would experience the greatest reduction in OCD symptoms and severity at post-intervention and 4-week follow-up"

Relationships, Doubt, and Obsessions (GGRO). However, as GGRO was designed to target symptoms of relationship OCD, the study samples were more selective, requiring all participants to have romantic relationship experience [29,30]. An RCT and an open pilot trial investigated the effectiveness of the apps OCfree and nOCD for OCD symptoms and found statistically significant reductions in OCD symptom severity at post-intervention [31,32]. However, app usage was guided or integrated alongside in-person therapy. Hence, it remains unclear whether self-guided mHealth apps for OCD are effective without therapist support. Furthermore, the studies utilized samples with clinically significant OCD, which might limit generalizability to populations with mild and subclinical OCD symptoms who are recommended to utilize self-guided, low-intensity treatments [33]. The authors also highlighted limitations, such as small sample sizes (N ≤ 50), the lack of a control group not undergoing CBT, and the necessity for replication in future RCTs. Other limitations include the absence of follow-up assessments in two out of four studies, and only one Asian sample being studied [30,32]. Overall, notable gaps in the literature regarding the effectiveness of self-guided mHealth app interventions for OCD in subclinical populations remain.
Moreover, variables affecting the efficacy of self-guided mHealth app programs for OCD are underexplored. Only Gamoran & Doron [34] examined moderators in a sample of 46,955 users of OCD.app. Initial OCD severity, age, gender, trait mood, and app usage data were identified as predictors of change. However, no psychological variables were analyzed as potential outcome moderators, leaving a gap in OCD treatment research [35]. A potential moderator of OCD outcomes from mHealth app programs is maladaptive perfectionism (MP). Perfectionism refers to one's tendency to demand and pursue unrealistically high standards of performance, accompanied by excessively critical self-evaluations [36]. Frost et al. [37] proposed a six-dimension measure of perfectionism, which involves being overly concerned about making mistakes (CM), setting and pursuing high personal standards (PS), perceiving high parental expectations (PE) and parental criticism (PC), doubting the quality of one's actions (DA), and preferring order and organization (O). As a multidimensional construct, perfectionism comprises adaptive and maladaptive aspects. While perfectionistic individuals seek excellence which may promote well-being [38], they may experience dysfunction when excessively fixating on mistakes and meeting external standards. Maladaptive perfectionism (MP) is commonly observed in individuals with OCD [39]. It is a risk factor for OCD development and has been viewed as an essential predisposing trait [40]. Additionally, MP has been shown to impede OCD treatment [41]. As moderators can help to identify individuals who may face a poor prognosis and assist in matching individuals with appropriate treatments, it would be useful to examine how baseline MP levels can affect intervention outcomes for self-guided CBT-based mHealth app programs for OCD."

"Using a randomized controlled design, we evaluated the efficacy of a self-guided program on a mHealth app in reducing subclinical OCD symptoms and severity amongst university students from Singapore. Additionally, we examined the association between MP and OCD symptom severity and investigated MP as a moderator of intervention outcomes. "

"The hypotheses of the study are in three parts. First, we expected participants in the OCD program to report significant reductions in OCD outcomes compared to the active control group at post-intervention and 4-week follow-up. Second, we hypothesized that baseline OCD symptom severity and MP will be positively correlated. Third, we predicted that participants with the lowest levels of MP would experience the greatest reduction in OCD symptoms and severity at post-intervention and 4-week follow-up."

METHODS

3a) Description of trial design (such as parallel, factorial) including allocation ratio

3b) Important changes to methods after trial commencement (such as eligibility criteria), with reasons

Does your paper address CONSORT subitem 3b? *

Copy and paste relevant sections from the manuscript (include quotes in quotation marks "like this" to indicate direct quotes from your manuscript), or elaborate on this item by providing additional information not in the ms, or briefly explain why the item is not applicable/relevant for your study

No important changes to methods after trial commencement

3b-i) Bug fixes, Downtimes, Content Changes

Bug fixes, Downtimes, Content Changes: ehealth systems are often dynamic systems. A description of changes to methods therefore also includes important changes made on the intervention or comparator during the trial (e.g., major bug fixes or changes in the functionality or content) (5-iii) and other "unexpected events" that may have influenced study design such as staff changes, system failures/downtimes, etc. [2].

	1	2	3	4	5	
subitem not at all important	(•)	( )	( )	( )	( )	essential

Clear selection

Does your paper address CONSORT subitem 4a? *

Copy and paste relevant sections from the manuscript (include quotes in quotation marks "like this" to indicate direct quotes from your manuscript), or elaborate on this item by providing additional information not in the ms, or briefly explain why the item is not applicable/relevant for your study

"The inclusion criteria required participants to be a Singapore Citizen or Permanent Resident, be able to read and understand English, be a student from the NUS between the ages of 18 and 30, present with mild or subclinical OCD symptoms, and own a mobile phone to download the Intellect app for use in the study. "

4a-i) Computer / Internet literacy

Computer / Internet literacy is often an implicit “de facto” eligibility criterion - this should be explicitly clarified.

	1	2	3	4	5	
subitem not at all important	○	○	○	○	◉	essential

Clear selection

Open vs. closed, web-based vs. face-to-face assessments: Mention how participants were recruited (online vs. offline), e.g., from an open access website or from a clinic, and clarify if this was a purely web-based trial, or there were face-to-face components (as part of the intervention or for assessment), i.e., to what degree got the study team to know the participant. In online-only trials, clarify if participants were quasi-anonymous and whether having multiple identities was possible or whether technical or logistical measures (e.g., cookies, email confirmation, phone calls) were used to detect/prevent these.

	1	2	3	4	5	
subitem not at all important	○	○	○	○	◉	essential

Clear selection

perfectionism.

Thereafter, participants were randomly allocated to the intervention or active control condition. To minimize potential confounding effects, participants were instructed not to use any other mHealth apps for the study duration. Additionally, to reduce demand characteristics, participants were not informed of the conditions' functions, or the study's objective to evaluate the efficacy of the OCD program. Instead, participants were informed that the study examined wellbeing and the learning effectiveness of mental health-related topics using a smartphone application. Participants were then instructed to download the Intellect app, create an account, and access the program of their allocated condition. Participants in the intervention group underwent 8 days of the OCD program, while participants in the active control group underwent 8 days of the cooperation program. Participation in each program took approximately five minutes per day. To encourage study adherence, participants were sent a daily reminder to complete app activities via WhatsApp Messenger or Telegram Messenger. To verify program completion, participants sent a screenshot of their learning logs to the investigator.

After verification, participants were sent a post-intervention survey link to complete the measures on OCD, psychological distress, and the App Engagement Scale (AES). Four weeks upon program completion, participants were sent a follow-up survey link to complete measures on OCD and psychological distress. To further control for demand effects and confounders, participants were asked to guess the study's hypothesis and indicate whether they had undergone mental health treatment during the past four weeks. Thereafter, they were debriefed on the study's objectives and hypotheses."

Does your paper address subitem 4a iii?

Copy and paste relevant sections from the manuscript (include quotes in quotation marks "like this" to indicate direct quotes from your manuscript), or elaborate on this item by providing additional information not in the ms, or briefly explain why the item is not applicable/relevant for your study

"Interested individuals signed up for the study by responding to the online advertisement via a link to an online survey hosted on Qualtrics. First, they completed a pre-screening test comprising measures on OCD and demographics and were screened for eligibility according to the inclusion criteria. Eligible participants read the Participation Information Sheet, and after providing informed consent, completed measures on psychological distress, and perfectionism. "

" Four weeks upon program completion, participants were sent a follow-up survey link to complete measures on OCD and psychological distress. To further control for demand effects and confounders, participants were asked to guess the study's hypothesis and indicate whether they had undergone mental health treatment during the past four weeks. Thereafter, they were debriefed on the study's objectives and hypotheses."

4b) Settings and locations where the data were collected

4b-i) Report if outcomes were (self-)assessed through online questionnaires

Clearly report if outcomes were (self-)assessed through online questionnaires (as common in web-based trials) or otherwise.

	1	2	3	4	5	
subitem not at all important	○	○	○	○	◉	essential

Clear selection

Does your paper address subitem 4b-i? *

Copy and paste relevant sections from the manuscript (include quotes in quotation marks "like this" to indicate direct quotes from your manuscript), or elaborate on this item by providing additional information not in the ms, or briefly explain why the item is not applicable/relevant for your study

"After verification, participants were sent a post-intervention survey link to complete the measures on OCD, psychological distress, and the App Engagement Scale (AES). Four weeks upon program completion, participants were sent a follow-up survey link to complete measures on OCD and psychological distress."

Does your paper address subitem 4b-ii?

Copy and paste relevant sections from the manuscript (include quotes in quotation marks "like this" to indicate direct quotes from your manuscript), or elaborate on this item by providing additional information not in the ms, or briefly explain why the item is not applicable/relevant for your study

not relevant to our study. there are no relevant institutional affiliations to our study.

5) The interventions for each group with sufficient details to allow replication, including how and when they were actually administered

5-i) Mention names, credential, affiliations of the developers, sponsors, and owners

Mention names, credential, affiliations of the developers, sponsors, and owners [6] (if authors/evaluators are owners or developer of the software, this needs to be declared in a “Conflict of interest” section or mentioned elsewhere in the manuscript).

	1	2	3	4	5	
subitem not at all important	○	○	○	○	◉	essential

Clear selection

evaluations (e.g., focus groups, usability testing), as these will have an impact on adoption/use rates and help with interpreting results.

	1	2	3	4	5	
subitem not at all important	○	○	○	○	◉	essential

Clear selection

investigated the effectiveness of the apps OCfree and nOCD for OCD symptoms and found statistically significant reductions in OCD symptom severity at post-intervention [31,32]. However, app usage was guided or integrated alongside in-person therapy. Hence, it remains unclear whether self-guided mHealth apps for OCD are effective without therapist support. Furthermore, the studies utilized samples with clinically significant OCD, which might limit generalizability to populations with mild and subclinical OCD symptoms who are recommended to utilize self-guided, low-intensity treatments [33]. The authors also highlighted limitations, such as small sample sizes (N ≤ 50), the lack of a control group not undergoing CBT, and the necessity for replication in future RCTs. Other limitations include the absence of follow-up assessments in two out of four studies, and only one Asian sample being studied [30,32]. Overall, notable gaps in the literature regarding the effectiveness of self-guided mHealth app interventions for OCD in subclinical populations remain. "

5-iii) Revisions and updating

Revisions and updating. Clearly mention the date and/or version number of the application/intervention (and comparator, if applicable) evaluated, or describe whether the intervention underwent major changes during the evaluation process, or whether the development and/or content was "frozen" during the trial. Describe dynamic components such as news feeds or changing content which may have an impact on the replicability of the intervention (for unexpected events see item 3b).

	1	2	3	4	5	
subitem not at all important	(●)	○	○	○	○	essential

Clear selection

information provided [1], if applicable.

	1	2	3	4	5	
subitem not at all important	○	○	○	○	○	essential

Does your paper address subitem 5-iv?

Copy and paste relevant sections from the manuscript (include quotes in quotation marks "like this" to indicate direct quotes from your manuscript), or elaborate on this item by providing additional information not in the ms, or briefly explain why the item is not applicable/relevant for your study

"ANCOVA is recommended for inferential testing intervention effects [75], as controlling for baseline scores ensures that results at post-intervention and follow-up are attributable to the intervention [76]"

"The assumptions for ANCOVA were met, except for the assumption for normality. However, due to the robustness of ANCOVA against deviations from normality when sample sizes are larger than 100 [79,80], analysis using ANCOVA proceeded."

Does your paper address subitem 5-v?

Copy and paste relevant sections from the manuscript (include quotes in quotation marks "like this" to indicate direct quotes from your manuscript), or elaborate on this item by providing additional information not in the ms, or briefly explain why the item is not applicable/relevant for your study

Yes. Screenshots and flowcharts have been uploaded through the submission system.

5-vi) Digital preservation

Digital preservation: Provide the URL of the application, but as the intervention is likely to change or disappear over the course of the years; also make sure the intervention is archived (Internet Archive, webcitation.org, and/or publishing the source code or screenshots/videos alongside the article). As pages behind login screens cannot be archived, consider creating demo pages which are accessible without login.

	1	2	3	4	5	
subitem not at all important	○	○	●	○	○	essential

Clear selection

Access: Describe how participants accessed the application, in what setting/context, if they had to pay (or were paid) or not, whether they had to be a member of specific group. If known, describe how participants obtained “access to the platform and Internet” [1]. To ensure access for editors/reviewers/readers, consider to provide a “backdoor” login account or demo mode for reviewers/readers to explore the application (also important for archiving purposes, see vi).

	1	2	3	4	5	
subitem not at all important	○	○	○	○	◉	essential

Clear selection

use any other mHealth apps for the study duration. Additionally, to reduce demand characteristics, participants were not informed of the conditions' functions, or the study's objective to evaluate the efficacy of the OCD program. Instead, participants were informed that the study examined wellbeing and the learning effectiveness of mental health-related topics using a smartphone application. Participants were then instructed to download the Intellect app, create an account, and access the program of their allocated condition."

5-viii) Mode of delivery, features/functionalities/components of the intervention and comparator, and the theoretical framework

Describe mode of delivery, features/functionalities/components of the intervention and comparator, and the theoretical framework [6] used to design them (instructional strategy [1], behaviour change techniques, persuasive features, etc., see e.g., [7, 8] for terminology). This includes an in-depth description of the content (including where it is coming from and who developed it) [1]," whether [and how] it is tailored to individual circumstances and allows users to track their progress and receive feedback" [6]. This also includes a description of communication delivery channels and – if computer-mediated communication is a component – whether communication was synchronous or asynchronous [6]. It also includes information on presentation strategies [1], including page design principles, average amount of text on pages, presence of hyperlinks to other resources, etc. [1].

	1	2	3	4	5	
subitem not at all important	○	○	○	○	◉	essential

Clear selection

were taught, while not overwhelming them. During the final program session, participants were guided to set goals for continual progress. Due to the challenging nature of ERP, the program also provided advice on concerns that are frequently encountered during ERP. An overview and timeline of the program are presented in Table 1 and Figure 2.

Cooperation Program (Active Control Condition)
Participants in the active control group were allocated to an 8-day self-guided program on cooperation. Having an active control group ensured that participants in both groups shared as many experiences as possible. This controlled for non-specific factors and attentional effects, which could impact the study's results [62]. The program aimed to improve participants' collaborative skills and interpersonal wellness through a series of content learning and practice exercises. To ensure that participants in both conditions spent a comparable amount of time and effort, the approximate duration for completing each daily session was matched to be five minutes."

5-ix) Describe use parameters

Describe use parameters (e.g., intended "doses" and optimal timing for use). Clarify what instructions or recommendations were given to the user, e.g., regarding timing, frequency, heaviness of use, if any, or was the intervention used ad libitum.

	1	2	3	4	5	
subitem not at all important	○	○	○	○	◉	essential

Clear selection

[illegible] expertise of professionals involved, if any, as well as “type of assistance offered, the timing and frequency of the support, how it is initiated, and the medium by which the assistance is delivered”. It may be necessary to distinguish between the level of human involvement required for the trial, and the level of human involvement required for a routine application outside of a RCT setting (discuss under item 21 – generalizability).

	1	2	3	4	5	
subitem not at all important	○	○	○	○	◉	essential

Clear selection

use any other mHealth apps for the study duration. Additionally, to reduce demand characteristics, participants were not informed of the conditions' functions, or the study's objective to evaluate the efficacy of the OCD program. Instead, participants were informed that the study examined wellbeing and the learning effectiveness of mental health-related topics using a smartphone application. Participants were then instructed to download the Intellect app, create an account, and access the program of their allocated condition. Participants in the intervention group underwent 8 days of the OCD program, while participants in the active control group underwent 8 days of the cooperation program. Participation in each program took approximately five minutes per day. To encourage study adherence, participants were sent a daily reminder to complete app activities via WhatsApp Messenger or Telegram Messenger. To verify program completion, participants sent a screenshot of their learning logs to the investigator.

After verification, participants were sent a post-intervention survey link to complete the measures on OCD, psychological distress, and the App Engagement Scale (AES). Four weeks upon program completion, participants were sent a follow-up survey link to complete measures on OCD and psychological distress. To further control for demand effects and confounders, participants were asked to guess the study's hypothesis and indicate whether they had undergone mental health treatment during the past four weeks. Thereafter, they were debriefed on the study's objectives and hypotheses.

"

Does your paper address subitem 5-xi? *

Copy and paste relevant sections from the manuscript (include quotes in quotation marks "like this" to indicate direct quotes from your manuscript), or elaborate on this item by providing additional information not in the ms, or briefly explain why the item is not applicable/relevant for your study

"To encourage study adherence, participants were sent a daily reminder to complete app activities via WhatsApp Messenger or Telegram Messenger. "

5-xii) Describe any co-interventions (incl. training/support)

Describe any co-interventions (incl. training/support): Clearly state any interventions that are provided in addition to the targeted eHealth intervention, as ehealth intervention may not be designed as stand-alone intervention. This includes training sessions and support [1]. It may be necessary to distinguish between the level of training required for the trial, and the level of training for a routine application outside of a RCT setting (discuss under item 21 – generalizability.

	1	2	3	4	5	
subitem not at all important	(●)	( )	( )	( )	( )	essential

Clear selection

and above were screened out due to a likely presence of clinically significant OCD [63]. The OCI-R showed acceptable internal consistency, with Cronbach's α of 0.68.

Frost Multidimensional Perfectionism Scale

The FMPS [37] is a 35-item scale which assesses perfectionism across 6 subscales: CM, PS, PE, PC, DA, and O. Items are rated on a 5-point Likert scale (1 = Strongly disagree; 5 = Strongly agree). The FMPS has demonstrated good internal consistency and convergent validity with other measures of perfectionism within non-clinical and clinical samples [37]. In this study, the MP dimension (FMPS-MP) was obtained by summing scores on the subscales of CM, DA, PE and PC [65]. The FMPS and FMPS-MP showed excellent internal consistency with Cronbach's α of 0.91 and 0.92, respectively.

Depression Anxiety Stress Scale-21

The DASS-21 [66] is a set of three scales assessing depression, anxiety, and stress, and is commonly used in student samples [67]. Each subscale contains seven items, which are rated on a 4-point Likert scale (0 = Did not apply to me at all; 3 = Applied to me very much or most of the time). In this study, the DASS-21 total score was computed by summing the scores of all items and used as a measure of general psychological distress [66]. Additionally, as depression, stress and anxiety have been identified in the pathogenesis and maintenance of OCD [69,70,71], baseline total DASS-21 scores were controlled for during analyses to disentangle the effect of the intervention on OCD symptoms from depression, stress, and anxiety. Internal consistency of the DASS-21 was excellent, with Cronbach's α of 0.91.

App Engagement Scale

The AES [72] is a 7-item scale which assesses app engagement. Each item is rated on a 5-point Likert scale (1 = Definitely disagree; 5 = Definitely agree). The total score is derived by summing all the items. Internal consistency of the AES is good, with Cronbach's α of 0.88.
"

Does your paper address subitem 6a-i?

Copy and paste relevant sections from manuscript text

The online questionnaires are self-report items that has been used by other web-based studies. CHERRIES items were not applied for our study

6a-ii) Describe whether and how “use” (including intensity of use/dosage) was defined/measured/monitored

Describe whether and how “use” (including intensity of use/dosage) was defined/measured/monitored (logins, logfile analysis, etc.). Use/adoption metrics are important process outcomes that should be reported in any ehealth trial.

	1	2	3	4	5	
subitem not at all important	○	○	○	○	◉	essential

Clear selection

Does your paper address subitem 6a-ii?

Copy and paste relevant sections from manuscript text

"To verify program completion, participants sent a screenshot of their learning logs to the investigator."

Does your paper address subitem 6a-iii?

Copy and paste relevant sections from manuscript text

Our paper did not obtain qualitative feedback.

6b) Any changes to trial outcomes after the trial commenced, with reasons

Does your paper address CONSORT subitem 6b? *

Copy and paste relevant sections from the manuscript (include quotes in quotation marks "like this" to indicate direct quotes from your manuscript), or elaborate on this item by providing additional information not in the ms, or briefly explain why the item is not applicable/relevant for your study

Not applicable. No changes to trial outcomes after trial commenced.

7a) How sample size was determined

NPT: When applicable, details of whether and how the clustering by care provides or centers was addressed

Does your paper address subitem 7a-i?

Copy and paste relevant sections from manuscript title (include quotes in quotation marks "like this" to indicate direct quotes from your manuscript), or elaborate on this item by providing additional information not in the ms, or briefly explain why the item is not applicable/relevant for your study

"Using Cohen's f = 0.10, alpha = .05 and power = .80, G*power 3.1 revealed a minimum number of 164 participants. Attrition rates in similar mHealth app-based studies conducted amongst NUS students have ranged from 1% to 44.9% [58,59,60]. Additionally, accounting for 10% of invalid data typically encountered in online research studies [61], this study aimed to recruit a minimum of 206 participants.
"

7b) When applicable, explanation of any interim analyses and stopping guidelines

Does your paper address CONSORT subitem 7b? *

Copy and paste relevant sections from the manuscript (include quotes in quotation marks "like this" to indicate direct quotes from your manuscript), or elaborate on this item by providing additional information not in the ms, or briefly explain why the item is not applicable/relevant for your study

Not applicable. No interim analyses and stopping guidelines are required for our study

8a) Method used to generate the random allocation sequence

NPT: When applicable, how care providers were allocated to each trial group

Does your paper address CONSORT subitem 8b? *

Copy and paste relevant sections from the manuscript (include quotes in quotation marks "like this" to indicate direct quotes from your manuscript), or elaborate on this item by providing additional information not in the ms, or briefly explain why the item is not applicable/relevant for your study

"In total, 1049 responses were screened. 225 participants met the eligibility criteria, completed the baseline survey, and were randomized to either the intervention or active control condition."

9) Mechanism used to implement the random allocation sequence (such as sequentially numbered containers), describing any steps taken to conceal the sequence until interventions were assigned

Does your paper address CONSORT subitem 9? *

Copy and paste relevant sections from the manuscript (include quotes in quotation marks "like this" to indicate direct quotes from your manuscript), or elaborate on this item by providing additional information not in the ms, or briefly explain why the item is not applicable/relevant for your study

This was not explicitly written in the manuscript, but simple randomisation procedures were performed on the Qualtrics platform.

11a) If done, who was blinded after assignment to interventions (for example, participants, care providers, those assessing outcomes) and how

NPT: Whether or not administering co-interventions were blinded to group assignment

11a-i) Specify who was blinded, and who wasn't

Specify who was blinded, and who wasn't. Usually, in web-based trials it is not possible to blind the participants [1, 3] (this should be clearly acknowledged), but it may be possible to blind outcome assessors, those doing data analysis or those administering co-interventions (if any).

	1	2	3	4	5	
subitem not at all important	○	○	◉	○	○	essential

Clear selection

11a-ii) Discuss e.g., whether participants knew which intervention was the "intervention of interest" and which one was the "comparator"

Informed consent procedures (4a-ii) can create biases and certain expectations - discuss e.g., whether participants knew which intervention was the "intervention of interest" and which one was the "comparator".

	1	2	3	4	5	
subitem not at all important	○	○	○	○	◉	essential

Clear selection

Does your paper address subitem 11a-ii?

Copy and paste relevant sections from the manuscript (include quotes in quotation marks "like this" to indicate direct quotes from your manuscript), or elaborate on this item by providing additional information not in the ms, or briefly explain why the item is not applicable/relevant for your study

"Social desirability bias is a concern because participant blinding was not fully feasible, given that the study's hypothesis might be guessed based on the content of the allocated program. However, participants who correctly guessed the study's hypothesis did not report significantly different results on OCD outcomes compared to incorrect participants, which gives us some confidence that the effect of social desirability bias was limited."

effort, the approximate duration for completing each daily session was matched to be five minutes."

12a) Statistical methods used to compare groups for primary and secondary outcomes

NPT: When applicable, details of whether and how the clustering by care providers or centers was addressed

Does your paper address CONSORT subitem 12a? *

Copy and paste relevant sections from the manuscript (include quotes in quotation marks "like this" to indicate direct quotes from your manuscript), or elaborate on this item by providing additional information not in the ms, or briefly explain why the item is not applicable/relevant for your study

Yes. Paragraphs on "Analytical approach" describes the statistical methods adopted.

Does your paper address subitem 12a-i? *

Copy and paste relevant sections from the manuscript (include quotes in quotation marks "like this" to indicate direct quotes from your manuscript), or elaborate on this item by providing additional information not in the ms, or briefly explain why the item is not applicable/relevant for your study

" As all missing data was verified to be missing at random, they were addressed using intent-to-treat (ITT) analyses [74], whereby missing data on the post-intervention and follow-up assessment were substituted with data from the last completed questionnaire. Missing AES data were replaced using the mean score. "

12b) Methods for additional analyses, such as subgroup analyses and adjusted analyses

Does your paper address CONSORT subitem 12b? *

Copy and paste relevant sections from the manuscript (include quotes in quotation marks "like this" to indicate direct quotes from your manuscript), or elaborate on this item by providing additional information not in the ms, or briefly explain why the item is not applicable/relevant for your study

Not applicable. No additional analyses were conducted in our study

Does your paper address subitem X26-i?

Copy and paste relevant sections from the manuscript (include quotes in quotation marks "like this" to indicate direct quotes from your manuscript), or elaborate on this item by providing additional information not in the ms, or briefly explain why the item is not applicable/relevant for your study

"The study's ethics was approved by the National University of Singapore's (NUS) Institutional Review Board (NUS-IRB-2023-444). The study was also pre-registered with ClinicalTrials.gov (registration number: NCT06202677)."

x26-ii) Outline informed consent procedures

Outline informed consent procedures e.g., if consent was obtained offline or online (how? Checkbox, etc.?), and what information was provided (see 4a-ii). See [6] for some items to be included in informed consent documents.

	1	2	3	4	5	
subitem not at all important	○	○	○	○	◉	essential

Clear selection

X26-iii) Safety and security procedures

Safety and security procedures, incl. privacy considerations, and any steps taken to reduce the likelihood or detection of harm (e.g., education and training, availability of a hotline)

	1	2	3	4	5	
subitem not at all important	○	○	○	○	◉	essential

Clear selection

Does your paper address subitem X26-iii?

Copy and paste relevant sections from the manuscript (include quotes in quotation marks "like this" to indicate direct quotes from your manuscript), or elaborate on this item by providing additional information not in the ms, or briefly explain why the item is not applicable/relevant for your study

this was not explicitly stated in the manuscript but the participation information sheet contained mental health hotlines for participants.

RESULTS

Yes. These information are clearly indicated on the Consort diagram and tables to the submission.

13b) For each group, losses and exclusions after randomisation, together with reasons

Does your paper address CONSORT subitem 13b? (NOTE: Preferably, this is shown in a CONSORT flow diagram) *

Copy and paste relevant sections from the manuscript (include quotes in quotation marks "like this" to indicate direct quotes from your manuscript), or elaborate on this item by providing additional information not in the ms, or briefly explain why the item is not applicable/relevant for your study

Yes. These information are clearly indicated on the Consort diagram and tables to the submission.

Does your paper address subitem 13b-i?

Copy and paste relevant sections from the manuscript or cite the figure number if applicable (include quotes in quotation marks "like this" to indicate direct quotes from your manuscript), or elaborate on this item by providing additional information not in the ms, or briefly explain why the item is not applicable/relevant for your study

Yes. These information are clearly indicated on the Consort diagram and tables to the submission.

14a) Dates defining the periods of recruitment and follow-up

Does your paper address CONSORT subitem 14a? *

Copy and paste relevant sections from the manuscript (include quotes in quotation marks "like this" to indicate direct quotes from your manuscript), or elaborate on this item by providing additional information not in the ms, or briefly explain why the item is not applicable/relevant for your study

No. the dates were not specified in the manuscript. However, data collection and recruitment occurred from 1 Jan 2024 to 30 March 2024.

Does your paper address subitem 14a-i?

Copy and paste relevant sections from the manuscript (include quotes in quotation marks "like this" to indicate direct quotes from your manuscript), or elaborate on this item by providing additional information not in the ms, or briefly explain why the item is not applicable/relevant for your study

Not applicable to our study- no critical secular events fell using study period.

14b) Why the trial ended or was stopped (early)

Does your paper address CONSORT subitem 14b? *

Copy and paste relevant sections from the manuscript (include quotes in quotation marks "like this" to indicate direct quotes from your manuscript), or elaborate on this item by providing additional information not in the ms, or briefly explain why the item is not applicable/relevant for your study

Not applicable. Trial did not end or stop early.

15) A table showing baseline demographic and clinical characteristics for each group

NPT: When applicable, a description of care providers (case volume, qualification, expertise, etc.) and centers (volume) in each group

divide issues, such as age, education, gender, social-economic status, computer/Internet/ehealth literacy of the participants, if known.

	1	2	3	4	5	
subitem not at all important	○	○	○	○	◉	essential

Clear selection

Does your paper address subitem 15-i? *

Copy and paste relevant sections from the manuscript (include quotes in quotation marks "like this" to indicate direct quotes from your manuscript), or elaborate on this item by providing additional information not in the ms, or briefly explain why the item is not applicable/relevant for your study

Yes. Participants' age and gender are included in the tables in the results segment.

16) For each group, number of participants (denominator) included in each analysis and whether the analysis was by original assigned groups

Does your paper address subitem 16-i? *

Copy and paste relevant sections from the manuscript (include quotes in quotation marks "like this" to indicate direct quotes from your manuscript), or elaborate on this item by providing additional information not in the ms, or briefly explain why the item is not applicable/relevant for your study

Our app program runs for 8-days with clear instructions of use for all participants. Segments on "OCD Program" and "Cooperation Program" define each of the program usage clearly.

16-ii) Primary analysis should be intent-to-treat

Primary analysis should be intent-to-treat, secondary analyses could include comparing only “users”, with the appropriate caveats that this is no longer a randomized sample (see 18-i).

	1	2	3	4	5	
subitem not at all important	○	○	○	○	◉	essential

Clear selection

17a) For each primary and secondary outcome, results for each group, and the estimated effect size and its precision (such as 95% confidence interval)

Does your paper address CONSORT subitem 17a? *

Copy and paste relevant sections from the manuscript (include quotes in quotation marks "like this" to indicate direct quotes from your manuscript), or elaborate on this item by providing additional information not in the ms, or briefly explain why the item is not applicable/relevant for your study

Yes. Effect sizes of the main analyses outcomes are included in table 3.

17a-i) Presentation of process outcomes such as metrics of use and intensity of use

In addition to primary/secondary (clinical) outcomes, the presentation of process outcomes such as metrics of use and intensity of use (dose, exposure) and their operational definitions is critical. This does not only refer to metrics of attrition (13-b) (often a binary variable), but also to more continuous exposure metrics such as "average session length". These must be accompanied by a technical description how a metric like a "session" is defined (e.g., timeout after idle time) [1] (report under item 6a).

	1	2	3	4	5	
subitem not at all important	(selected)	○	○	○	○	essential

Clear selection

Does your paper address CONSORT subitem 17b? *

Copy and paste relevant sections from the manuscript (include quotes in quotation marks "like this" to indicate direct quotes from your manuscript), or elaborate on this item by providing additional information not in the ms, or briefly explain why the item is not applicable/relevant for your study

Not applicable. Outcomes are not binary in this study.

18) Results of any other analyses performed, including subgroup analyses and adjusted analyses, distinguishing pre-specified from exploratory

Table 4."

18-i) Subgroup analysis of comparing only users

A subgroup analysis of comparing only users is not uncommon in ehealth trials, but if done, it must be stressed that this is a self-selected sample and no longer an unbiased sample from a randomized trial (see 16-iii).

	1	2	3	4	5	
subitem not at all important	(•)	( )	( )	( )	( )	essential

Clear selection

Does your paper address subitem 18-i?

Copy and paste relevant sections from the manuscript (include quotes in quotation marks "like this" to indicate direct quotes from your manuscript), or elaborate on this item by providing additional information not in the ms, or briefly explain why the item is not applicable/relevant for your study

"Not applicable. Subgroup analysis of comparing only users not conducted."

19) All important harms or unintended effects in each group

(for specific guidance see CONSORT for harms)

[illegible]
problems, and other unexpected/unintended incidents. “Unintended effects” also includes unintended positive effects [2].

	1	2	3	4	5	
subitem not at all important	○	○	○	○	◉	essential

Clear selection

Does your paper address subitem 19-i?

Copy and paste relevant sections from the manuscript (include quotes in quotation marks "like this" to indicate direct quotes from your manuscript), or elaborate on this item by providing additional information not in the ms, or briefly explain why the item is not applicable/relevant for your study

The participant information sheet that participants read and consented as part of the study (segment on "Procedure and Participant Flow" in the paper) addressed privacy and confidentiality concerns, as well as who to contact should they require assistance.

Does your paper address subitem 19-ii?

Copy and paste relevant sections from the manuscript (include quotes in quotation marks "like this" to indicate direct quotes from your manuscript), or elaborate on this item by providing additional information not in the ms, or briefly explain why the item is not applicable/relevant for your study

Not applicable. Qualitative feedback was not obtained in our study.

DISCUSSION

22) Interpretation consistent with results, balancing benefits and harms, and considering other relevant evidence

NPT: In addition, take into account the choice of the comparator, lack of or partial blinding, and unequal expertise of care providers or centers in each group

Does your paper address subitem 22-i? *

Copy and paste relevant sections from the manuscript (include quotes in quotation marks "like this" to indicate direct quotes from your manuscript), or elaborate on this item by providing additional information not in the ms, or briefly explain why the item is not applicable/relevant for your study

Yes. "Discussion" segment of the paper described these information.

22-ii) Highlight unanswered new questions, suggest future research

Highlight unanswered new questions, suggest future research.

	1	2	3	4	5	
subitem not at all important	○	○	○	○	◉	essential

Clear selection

Does your paper address subitem 22-ii?

Copy and paste relevant sections from the manuscript (include quotes in quotation marks "like this" to indicate direct quotes from your manuscript), or elaborate on this item by providing additional information not in the ms, or briefly explain why the item is not applicable/relevant for your study

"Future research should explore longer follow-up periods and broader populations."

subitem not at all important ○ ○ ○ ○ ◉ essential

Clear selection

Does your paper address subitem 20-i? *

Copy and paste relevant sections from the manuscript (include quotes in quotation marks "like this" to indicate direct quotes from your manuscript), or elaborate on this item by providing additional information not in the ms, or briefly explain why the item is not applicable/relevant for your study

"However, our study had several limitations. First, data regarding how long participants spent applying skills from the programs in their daily lives were not collected. CBT research has found that greater application of CBT skills improves treatment outcomes [95], hence it is probable that participants who practiced the skills taught in our programs more frequently would have made increased gains. Moreover, there was no objective assessment of participant engagement and effort expended on the intervention programs as the time participants spent using the app was not tracked or controlled for. "

21) Generalisability (external validity, applicability) of the trial findings

NPT: External validity of the trial findings according to the intervention, comparators, patients, and care providers or centers involved in the trial

Does your paper address subitem 21-i?

Copy and paste relevant sections from the manuscript (include quotes in quotation marks "like this" to indicate direct quotes from your manuscript), or elaborate on this item by providing additional information not in the ms, or briefly explain why the item is not applicable/relevant for your study

"Given that participants with clinically significant levels of OCD were excluded from our sample, our study's participants represent a narrower segment of the OCD severity spectrum than the general, broader OCD population. "

"Finally, as participants were recruited from one university and comprised mostly of female students, the external validity of our study may be limited. Future studies may replicate our findings with a more diverse sample of young adults. "

21-ii) Discuss if there were elements in the RCT that would be different in a routine application setting

Discuss if there were elements in the RCT that would be different in a routine application setting (e.g., prompts/reminders, more human involvement, training sessions or other co-interventions) and what impact the omission of these elements could have on use, adoption, or outcomes if the intervention is applied outside of a RCT setting.

	1	2	3	4	5	
subitem not at all important	(●)	( )	( )	( )	( )	essential

Clear selection

23) Registration number and name of trial registry

Does your paper address CONSORT subitem 23? *

Copy and paste relevant sections from the manuscript (include quotes in quotation marks "like this" to indicate direct quotes from your manuscript), or elaborate on this item by providing additional information not in the ms, or briefly explain why the item is not applicable/relevant for your study

Trial Registration: ClinicalTrials.gov NCT06202677

24) Where the full trial protocol can be accessed, if available

Does your paper address CONSORT subitem 24? *

Cite a Multimedia Appendix, other reference, or copy and paste relevant sections from the manuscript (include quotes in quotation marks "like this" to indicate direct quotes from your manuscript), or elaborate on this item by providing additional information not in the ms, or briefly explain why the item is not applicable/relevant for your study

Not applicable. The authors of this study can be contacted for further queries pertaining to this study.

the decision to publish the study are entirely independent of Intellect Pte Ltd.

X27) Conflicts of Interest (not a CONSORT item)

X27-i) State the relation of the study team towards the system being evaluated

In addition to the usual declaration of interests (financial or otherwise), also state the relation of the study team towards the system being evaluated, i.e., state if the authors/evaluators are distinct from or identical with the developers/sponsors of the intervention.

	1	2	3	4	5	
subitem not at all important	○	○	○	○	◉	essential

Clear selection

Does your paper address subitem X27-i?

Copy and paste relevant sections from the manuscript (include quotes in quotation marks "like this" to indicate direct quotes from your manuscript), or elaborate on this item by providing additional information not in the ms, or briefly explain why the item is not applicable/relevant for your study

The study was partly funded by Intellect Pte Ltd. OS holds the position of Vice President of Clinical at Intellect Pte Ltd; he also holds equity in Intellect.

What were the most important changes you made as a result of using this checklist?

Your answer

How much time did you spend on going through the checklist INCLUDING making changes in your manuscript *

approximately 4 to 5 hours

As a result of using this checklist, do you think your manuscript has improved? *

- (x) yes
- ( ) no
- ( ) Other:

Any other comments or questions on CONSORT EHEALTH

Your answer

STOP - Save this form as PDF before you click submit

To generate a record that you filled in this form, we recommend to generate a PDF of this page (on a Mac, simply select "print" and then select "print as PDF") before you submit it.

When you submit your (revised) paper to JMIR, please upload the PDF as supplementary file.

Don't worry if some text in the textboxes is cut off, as we still have the complete information in our database. Thank you!

Final step: Click submit !

Click submit so we have your answers in our database!

Submit

Clear form

Never submit passwords through Google Forms.

This content is neither created nor endorsed by Google. Report Abuse - Terms of Service - Privacy Policy

Google Forms
